# Cardioprotective Potential of Aqueous Extract of *Fumaria indica* on Isoproterenol-Induced Myocardial Infarction in SD Rats

**DOI:** 10.1155/2022/2112956

**Published:** 2022-05-31

**Authors:** Aimen Sajid, Taseer Ahmad, Muhammad Ikram, Taous Khan, Abdul Jabbar Shah, Mater H. Mahnashi, Abdulaziz Hassan Alhasaniah, Ahmed Abdullah Al Awadh, Ibrahim Abdullah Almazni, Mohammed Merae Alshahrani

**Affiliations:** ^1^Cardiovascular Research Group, Department of Pharmacy, COMSATS University Islamabad, Abbottabad Campus, University Road, Abbottabad 22060, KPK, Pakistan; ^2^Department of Pharmacology, College of Pharmacy, University of Sargodha, University Road, Sargodha, Punjab 40100, Pakistan; ^3^Department of Pharmaceutical Chemistry, College of Pharmacy, Najran University, Najran, Saudi Arabia; ^4^Department of Clinical Laboratory Sciences, Faculty of Applied Medical Sciences, Najran University, 1988, Najran 61441, Saudi Arabia

## Abstract

Ischemic heart disease (IHD) treatments and preventions by using plant extract and its phytochemical constituents have achieved considerable attention globally due to its cardioprotective effects. This study is aimed at investigating the cardioprotective and vascular effects of *Fumaria indica* (*F. indica*) crude extract on isoproterenol- (ISO-) induced myocardial infarction (MI) in Sprague-Dawley (SD) rats. Rats treated with isoproterenol (85 mg/kg, s.c), administered. Twice at an interval of 24 h showed a significant ST-segment elevation in ECG, edema, and necrosis in histopathology and also in troponin I (cTnI), creatine phosphokinase (CPK), lactate dehydrogenase (LDH), and aspartate aminotransferase (AST). Pretreatment with *F. indica* (10, 30, and 100 mg/kg, p.o) for 21 days significantly reversed the effects of isoproterenol-induced ischemic changes in the ECG, levels of cTnI, CPK, LDH, and AST, and histopathological changes. In isolated rat atrial strips, *F. indica* induced negative chronotropic and inotropic effects which were not affected by pretreatment with atropine, excluding role of cardiac muscarinic receptors. Cumulative addition of the extract induced a vasorelaxant effect on phenylephrine-evoked contractions in isolated rat aortic rings, which remained unchanged when challenged with _L_-NAME, excluding role of endothelial NO. However, extract of *F. indica* concentration dependently reversed contractions evoked with high K^+^, indicating calcium entry blocking effect. In conclusion, the *F. indica* extract is a cardioprotective remedy that ameliorates the isoproterenol-induced cardiotoxic effects and reverses cardiac ischemia, and the calcium antagonistic effect might be of useful in the treatment of MI.

## 1. Introduction

Cardiovascular diseases (CVDs) are the leading cause of chronic disability and premature death for all regions of the world, and ischemic heart disease (IHD) accounts for the majority of health lost to CVDs. Myocardial infarction (MI), also called heart attack, is the acute necrotic condition of the myocardium that occurs due to myocardial demand and coronary blood supply mismatch [1]. Coronary atherosclerosis with activated inflammation in the vascular wall may develop MI. Apart from the mechanisms mediating coronary artery occlusion and supply-demand mismatch, reactive oxygen species (ROS) play vital role in the injury following MI [2].

Although conventional medicine in cardiovascular disorders is effective but potentially associated serious adverse effects, expensive lifelong management and rebound phenomenon are still key challenges [3]. A wide array of plants and active principles with minimum side effects provide an alternative therapy for ischemic heart diseases such as *Cichorium intybus* [4], *Ginkgo biloba* [5], *Nerium oleander* Linn [6], and *Terminalia arjuna* [7].

*Fumaria indica* L. (*F. indica*) synonyms: *F. parviflora* and *F. vaillantii* belong to family “Papaveraceae” commonly found in plains and lower hills of Pakistan, Turkey, India, Afghanistan, Iran, and Central Asia. *F. indica* commonly known as Fumitory, Fumus, and Earth smoke in English and locally known as “Murghipal” and “Papra” [8]. Ethnomedicinal studies have gained much importance in recent years, bringing to lights the numerous little unknown and known medicinal uses especially of plant origins. *F. indica* has a long history of use as a blood purifier [9] and antihypertensive remedy [10] in traditional medicine and has been reported to possess hepatoprotective [11], spasmolytic and spasmogenic [12], antibacterial [13], anti-inflammatory [14], gastroprotective [15], antidengue [16], antidiabetic, antileishmanial [17], and antioxidant activity [18, 19]. Compounds that are responsible for these activities are mostly isoquinolinic alkaloids, among which protopine is the most frequently found [20]. Previous studies have shown that protopine possesses antihypertensive [21], antiarrhythmic [22], and smooth muscle relaxant effect [23]. Protopine also exhibits calcium channel antagonist activity [24]. Beside isoquinolinic alkaloids, *F. indica* also contains polyphenols and flavonoids [17]. Phenolics and flavonoids have ideal structural chemistry for quenching free radicals which make them imperative in fighting and preventing conditions like oxidative stress, cell death, and cytotoxicity [25]. Antioxidant and calcium channel blocking activity provides protective effects in ischemic heart diseases [26, 27]. However, the literature lacks investigation of *F. indica* in cardiac ischemia so the current study investigated the *F. indica* extract for its cardioprotective potential and its effect on isolated cardiac tissue and aorta.

## 2. Materials and Methods

### 2.1. Experimental Animals

Experiments were performed in accordance with the rulings of the Institute of Laboratory Animal Resources, Commission on Life sciences, National Research Council [28] and approved by the Ethical Committee of Pharmacy Department, CUI, Abbottabad Campus, Pakistan. Sprague-Dawley (SD) rats 200-250 g weight were kept at the animal house of the CUI, Abbottabad Campus, maintained at (23-25°C) under 12-hour light/dark cycle allowed with food and water *ad libitum*.

### 2.2. Drugs and Standards

Drugs used in this study were purchased from the source specified: pentothal sodium (Abbott Laboratories, Pakistan), isoproterenol hydrochloride, atropine sulfate, phenylephrine hydrochloride, potassium chloride, atenolol, and N*ω*-nitro-L-arginine methyl ester hydrochloride (_L_-NAME) (Sigma Chemicals Company, USA). Analytical grade chemicals were used. All chemical stock solutions were made in distilled water and normal saline.

### 2.3. Extraction

The collected *F. indica* was rinsed with tap water to remove the associated dust and shade dried at ambient temperature (24-26°C) with occasional rotation. The dried material was grinded to obtain 1.5 kg of dried powder. The powdered material was then subjected to extraction using methanol as menstrum at ambient temperature. The soaked materials were stirred daily with stainless steel rod in order to avoid caking and to get uniform mixing. The soaking was performed for 21, 7, and 3 days, successively in order to fully exhaust the plant materials. Whatman filter paper and muslin cloth were used in order to remove the solid particles. The filtration was done using rotary evaporator and water bath set at 40°C. To obtain the final percentage yield of crude dried extract of *F. indica*, crude extract was dried in a fume hood [29].

### 2.4. Isoproterenol-Induced Myocardial Infarction Rat Model

SD rats were divided in to 6 groups having 5 rats in each. Group 1 served as control and was kept on normal saline. Group 2 received isoproterenol (85 mg/kg, s.c) on days 20 and 21. Groups 3–5 received 10, 30, and 100 mg/kg (p.o) respective doses of extract of *F. indica* for three weeks and isoproterenol (85 mg/kg, s.c) only on days 20 and 21. Group 6 received atenolol (10 mg/kg, s.c) for 3 weeks and isoproterenol (85 mg/kg, s.c) only on days 20 and 21. Twelve hours after isoproterenol last dose, all the rats were anesthetized with thiopental sodium (40-90 mg/kg, i.p) and ECG was recorded; biochemical parameters were identified, and histopathological study was also carried out.

### 2.5. Electrocardiography (ECG)

ECG measurement of SD rats was carried out in anesthetized rats, placed in a supine position from all the groups. After 30 min of complete anesthesia induction, acupuncture needle electrodes was inserted subcutaneously according to the lead II (right foreleg, left rear leg, and left foreleg) as per ECG scheme. ECG was recorded for 1 minute every 5 minutes in anesthetized rats by using a PowerLab connected with BioAmp and analyzed by the LabChart 7 software (ADInstrument, Australia). Each channel was amplified and sampled at a rate of 2 KHz and 5 mV range of a high-pass filter setting of 1 Hz. Alterations in ECG pattern (ST-segment depression or elevation) in experimental and normal rats were recorded and analyzed [30].

### 2.6. Biochemical Estimations

For biochemical estimation, serum was separated by centrifugation and cardiac markers such as troponin I (cTnI), creatine phosphokinase (CPK), lactate dehydrogenase (LDH), and aspartate aminotransferase (AST) which were estimated according to the procedure reported by [31–33].

### 2.7. Histopathological Examination

SD rats from all groups were sacrificed, and removed hearts were immediately fixed in buffered formalin (10%). Heart ventricular mass from apex to the base was sectioned and embedded (with paraffin) after dehydration with alcohol and xylene clearance. Staining of 5 *μ*m thick histological sections was done with eosin and hematoxylin after obtaining from the paraffin blocks which were examined under light microscope.

### 2.8. Myocardial Infarction Determination by Direct Staining

Rat hearts were removed and frozen immediately, and from the apex towards the atrioventricular (AV) groove, thick slice of 3-5 mm was cut. The slice was incubated in 1% solution of 2,3,5-triphenyltetrazolium chloride (in phosphate buffer saline pH 7.4) for 20 min at 37°C. Examination of the sections was done under light, and photographs were taken [30].

### 2.9. Underlying Mechanism on Isolated Rat Atria

As described previously [34, 35], rats were sacrificed by cervical dislocation and atria were removed and mounted in tissue bath containing Kreb's solution, aerated with carbogen gas (5% CO_2_ in 95% O_2_) at 32°C. 1 g resting tension was applied to each tissue, and equilibrium period of 10-15 min was allowed before studying the effect of different concentrations of extract of *F. indica* on spontaneous atrial contractions. The tissues were preincubated with atropine (1 *μ*M) for 30 min to explore underlying mechanism. Changes in isometric tension of the atria via a force-displacement transducer connected to PowerLab Data Acquisition System were measured.

### 2.10. Vascular Effects in Isolated Rat Aorta

As described previously [36, 37], the descending thoracic aorta was isolated from the rats, transferred into the petri plate containing normal Kreb's solution, and cleaned. Kreb's solution was composed of the following (mM): glucose 11.7 (pH 7.4), KCL 4.7, NaHCO_3_ 25.0, NaCl 118.2, CaCl_2_ 2.5, MgSO_4_ 1.2, and KH_2_PO_4_ 1.3. In tissue bath containing carbogen-aerated Kreb's solution, aortic ring (3 mm) preparations were carefully hanged and attached to a force transducer, which was coupled with PowerLab Data Acquisition System (ADInstruments, Sydney, Australia). Stabilization period of 30-45 min was given to each aortic ring with a preload of 2 g by changing buffer every 15 min. Sustained contractions were induced by phenylephrine, and the cumulative addition of different concentrations (0.1–10 *μ*g/mL) of the extract was made to determine effect on vascular tone. _L_-NAME (10 *μ*M) was used to determine role of vascular nitric oxide.

### 2.11. Statistical Analysis

All the values were expressed as mean ± S.D. One-way ANOVA followed by Tukey's test was used to test statistical significance through the GraphPad Prism software (GraphPad, San Diego, CA, USA). Differences were considered to be statistically significant when *p* < 0.05.

## 3. Results

### 3.1. Effect of *F. Indica* Extract on Electrocardiography (ECG) Parameters

**Figure 1:**
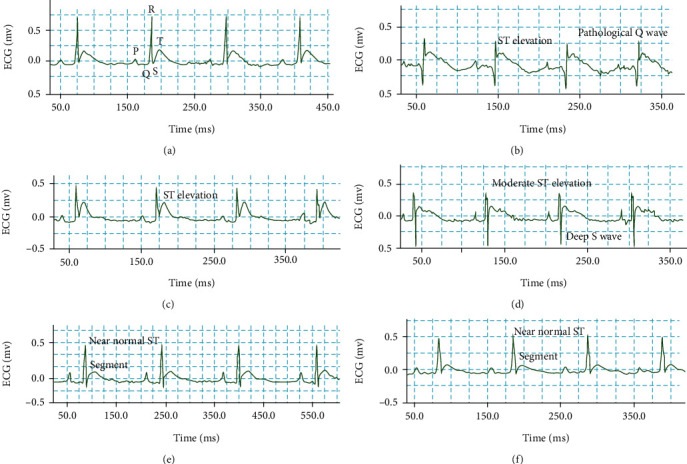
Representative electrocardiogram (ECG) tracing of (a) control, (b) isoproterenol, (c) *F. indica* crude extract 10 mg/kg + ISO, (d) 30 mg/kg + ISO, (e) 100 mg/kg + ISO, and (e) group 6 atenolol (10 mg/kg) + ISO (f) treated rats (recorded from lead II with recording speed 50 ms/div). mv: millivolt; ms: millisecond.

**Figure 2:**
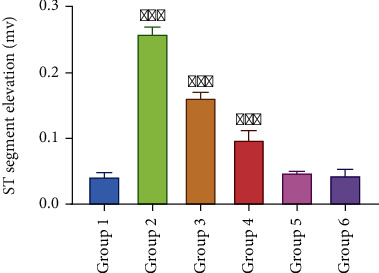
ST-segment changes of control and experimental animals. Group 1, control showed normal ST segment. Group 2, isoproterenol-treated showed significant ST-segment elevation. Group 3, *F. indica* crude extract 10 mg/kg + ISO. Group 4, *F. indica* 30 mg/kg + ISO. Group 5, *F. indica* 100 mg/kg + ISO showed marked reduction in ST elevation. Group 6, atenolol (10 mg/kg) + ISO-treated rats represent maximum reduction in ST-segment elevation. Values are expressed as mean ± S.D. *n* = 6. Compared with control, ^∗∗∗^*p* < 0.001, which represents the significant difference vs. control.

Normal control rats were kept on normal diet, and drinking water showed no changes in the ECG pattern and ST segment (Figures 1(a) and 2), while isoproterenol-treated rats showed pathologic Q wave formation and significant ST-segment elevation (Figures 1(b) and 2). Restoration of normal ECG pattern occurred in atenolol (10 mg/kg) and *F. indica* extract (10, 30, and 100 mg/kg)-pretreated group in a dose-dependent manner.

### 3.2. Effect of *F. Indica* Crude Extract on Cardiac Marker Enzymes

**Figure 3:**
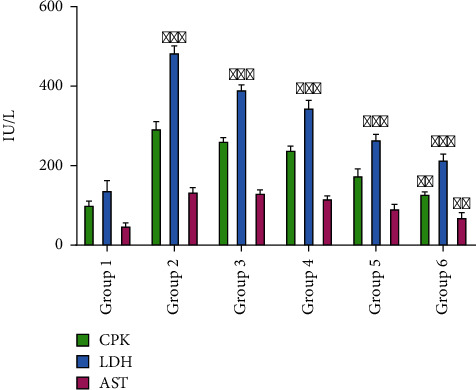
Changes in the levels of creatine phosphokinase (CPK), lactate dehydrogenase (LDH), and aspartate transaminase (AST). Group 1, control; group 2, isoproterenol-induced MI group; group 3, *F. indica* extract-treated group (10 mg/kg); group 4, *F. indica* extract-treated group (30 mg/kg); group 5, *F. indica* extract-treated group (100 mg/kg); group 6, atenolol-treated group (10 mg/kg). Values are expressed as mean ± S.D. *n* = 6. Compared with control, ^∗∗^*p* < 0.01 and ^∗∗∗^*p* < 0.001, which represent the significant difference vs. control.

Qualitative estimation of cTnI in all rats of the control group was negative (Table 1), and the serum CPK, LDH, and AST concentrations were 101 ± 2.97, 139.5 ± 4.75, and 49.8 ± 2.67 IU/L as shown in Figure 3. Rats treated with isoproterenol showed positive cTnI and serum CPK 294.6 ± 3.76, LDH 485.6 ± 5.63, and AST 135 ± 3.99 IU/L as shown in Figure 3. Significant decreased in number of positive cTnI test cases (Table 1) and serum level of CPK 263.2 ± 2.67, LDH 392.6 ± 4.57, and AST 134.2 ± 5.56 IU/L (Figure 3) were observed in rats pretreated with *F. indica* (10 mg/kg). Rats pretreated with *F. indica* crude extract (30 mg/kg) showed less positive cTnI test cases (Table 1), and a significant serum reduction level of CPK 240.6 ± 3.97, LDH 347.7 ± 5.57, and AST 118.8 ± 4.36 IU/L (Figure 3) was observed. Pretreatment with *F. indica* crude extract (100 mg/kg) restored significantly the serum cardiac biomarker level alteration induced by ISO, such as significant decreased in the positive cTnI test cases (Table 1) and serum level of CPK 176.6 ± 2.97, LDH 267.7 ± 4.37, and AST 93.6 ± 3.69 IU/L (Figure 3). In addition, pretreatment with atenolol (10 mg/kg) also significantly decreased positive cTnI test cases and the serum level of the CPK 130.4 ± 1.67, LDH 216.0 ± 3.58, and AST 71.8 ± 3.97 IU/L (Figure 3).

### 3.3. Effect of *F. Indica* Crude Extract on Histopathology and TTC Staining

**Figure 4:**
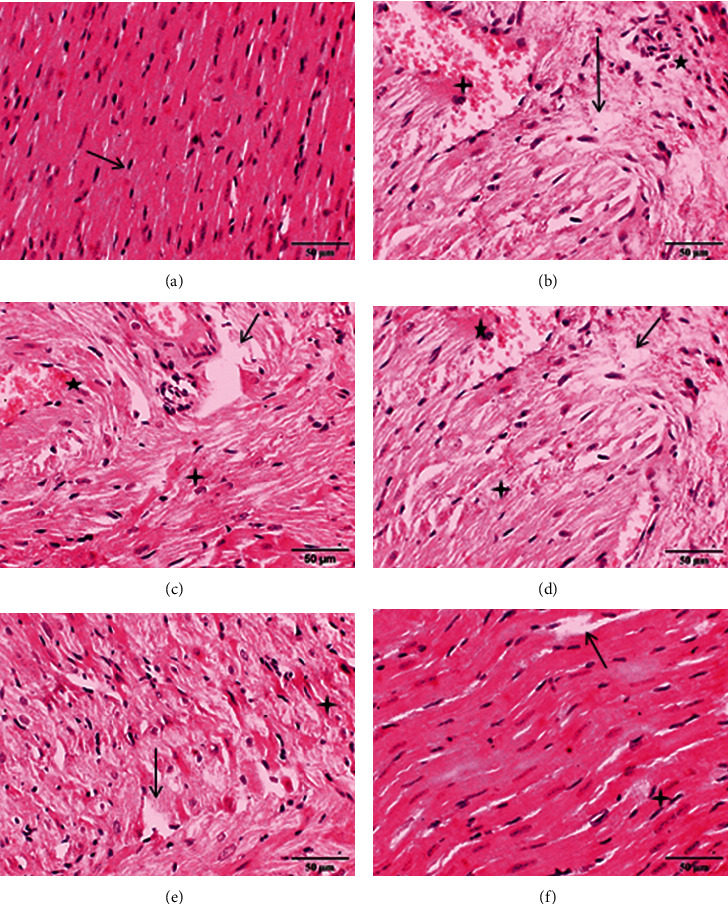
Effect of *F. indica* extract pretreatment on histopathological changes. (a) Heart tissue of a normal control showed normal cardiomyocytes (arrow) with one centrally placed nucleus. (b) The isoproterenol- (ISO-) treated group showed marked edema (arrow), inflammatory cell infiltration (star), and necrosis (four-point star). (c) Pretreatment with *F. indica* extract (10 mg/kg) + ISO showed minimum reduction of edema, inflammatory cell infiltration, and necrosis. (d) *F. indica* extract (30 mg/kg) + ISO produced moderate reduction of edema, inflammatory cell infiltration, and necrosis. (e) *F. indica* extract (100 mg/kg) + ISO produced maximum reduction of edema, inflammatory cell infiltration, and necrosis. (f) Atenolol (10 mg/kg) + ISO showed moderate reduction of edema, inflammatory cell infiltration, and necrosis.

**Figure 5:**
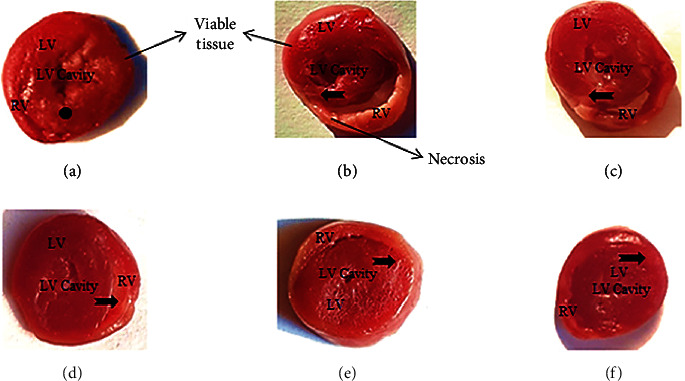
Effect of *F. indica* extract on representative photomicrograph of heart tissues dyed with 2,3,5-triphenyltetrazolium chloride. (a) The control group showed stained brick red region (dot), an indicator of viable tissue, (b) the isoproterenol- (ISO-) induced MI group showed marked unstained region (notched arrow), an indicator of total necrosis, (c) *F. indica* extract 10 mg/kg + ISO showed mild decrease in unstained region, (d) *F. indica* extract 30 mg/kg + ISO showed moderate decrease in unstained region, (e) *F. indica* extract 100 mg/kg + ISO showed marked decrease in unstained region, and (f) atenolol 10 mg/kg + ISO showed moderate decrease in unstained region. LV: left ventricle; RV: right ventricle.

In histopathological examination of the control group, normal architecture was observed (Figure 4(a)), whereas ISO-treated rats showed edema, inflammatory cell infiltration, and necrosis (Figure 4(b)). The animals pretreated with *F. indica* crude extract (Figures 4(c)–4(e)) showed much less intensity and distribution of edema, inflammatory cell infiltrations, and necrosis. TTC-stained brick red (dark region), an indicator of mitochondrial respiration, was observed in the heart tissues of the control (Figure 5(a)) and *F. indica* crude extract (10, 30, and 100 mg/kg)-treated group. In the ISO-treated group (Figure 5(b)), the unstained region indicates total necrosis. The heart tissue sections pretreated with *F. indica* crude extract (10, 30, and 100 mg/kg) (Figures 5(c)–5(e)) as compared with the ISO-treated group showed a lesser degree of unstained region.

### 3.4. *In Vitro* Rat Right Atrial Study

**Figure 6:**
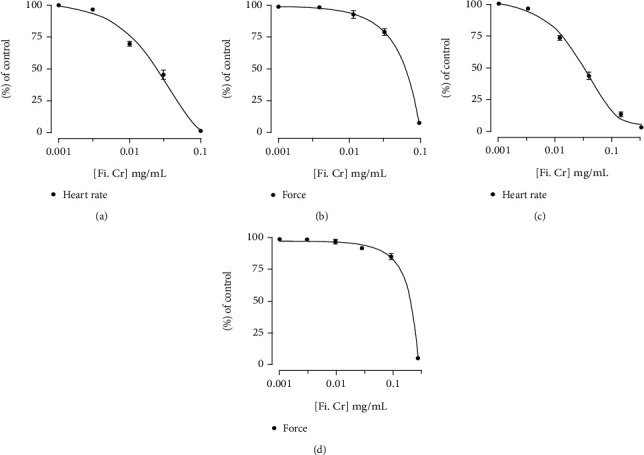
Concentration response curves show the effect of *F. indica* crude extract on heart rate and force of contraction in isolated SD rat right atrial preparations in the (a, b) absence and (c, d) presence of atropine (1 *μ*M). Values shown are mean ± S.D. *n* = 6.

Right atrial strips from normal rats were used to investigate chronotropic and inotropic effects of *F. indica*. The crude extract has shown dose-dependent decrease in force of contraction (100%) and heart rate (100%) (Figures 6(a) and 6(b)). In atropine-pretreated tissues, decrease in force of contraction and heart rate was shown to be 100%, respectively (Figures 6(c) and 6(d)).

### 3.5. *In Vitro* Vascular Reactivity Studies

**Figure 7:**
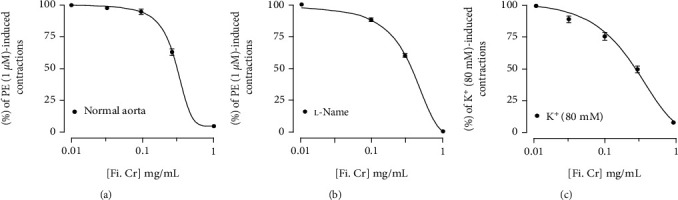
. Vasorelaxant effect of *F. indica* crude extract (a) without and (b) with _L_-NAME (10 *μ*M) pretreated normal rat aorta against phenylephrine-induced contractions, and (c) shows vasorelaxant effect of *F. indica* crude extract on high K^+^ (80 mM)-induced contractions in normal rat aortic preparation. Values shown are mean ± S.D. *n* = 6.

To perform vascular reactivity studies, aortae isolated from normal rats were used. The cumulative addition of *F. indica* crude extract against phenylephrine precontractions produced relaxation with EC_50_ value of 0.1 mg/mL (0.1-0.2) (Figure 7(a)). _L_-NAME (10 *μ*M)-pretreated rings showed relaxation with EC_50_ value of 0.10 mg/mL (0.1-0.2) (Figure 7(b)) precontracted with PE (1 *μ*M). High K^+^-contracted tissues showed complete relaxation with *F. indica* crude extract (Figure 7(c)).

## 4. Discussion

Isoproterenol (ISO), a synthetic nonselective *β*-adrenoceptor agonist, has been found to induce acute MI in rats at high doses. Isoproterenol leads to myocardial hyperfunction due to increase both in chronotropism and inotropism leading to imbalance between oxygen demand and supply to cardiomyocytes [38]. Isoproterenol results in increase in intracellular calcium level and oxidative stress through activation of cyclic adenosine monophosphate (cAMP) signaling pathway [39]. Isoproterenol results in the generation of highly cytotoxic oxygen derived free radicals which cause lipid membrane peroxidation, leading to both structural and functional myocardial cell injury and death [40]. The pathophysiological changes following isoproterenol administration are comparable to those taking place in human myocardial alterations [33].

For the definite MI diagnosis, evolving pattern of ECG abnormalities is the main criterion generally used [41]. In isoproterenol-induced rats significant ECG pattern alteration were observed when compared with normal rats. High dose of isoproterenol caused myocardial necrosis due to severe stress in the myocardium, deep Q wave and ST elevation in ECG, and structural and conduction disturbances in the heart. The typical finding was the ST-segment elevation which is an indicative sign of acute ischemic tissue injury in the transmural region of myocardium [42]. Isoproterenol-induced increased ROS and calcium influx into the myocardium result in myocyte necrosis and the collapse of cell membrane permeability [43]. Increase ROS production results in increased mitochondrial membrane permeability, outer membrane rupture, and the release of apoptotic signaling molecules [44]. Activation of calcium-dependent phospholipases, proteases enzymes, and high energy phosphate depletion by calcium overload affects the membrane [45, 46]. ECG abnormalities such as formation of pathological Q wave, ST-segment elevation, and conduction disturbances in isoproterenol-treated rats might be characterized by the consecutive loss of function of the ischemic myocardial cell membrane [47]. *F. indica* extract pretreatment reversed the ISO-induced altered ECG patterns, suggesting membrane protective effect, which was dose-dependent. This membrane protective effect might be due to the antioxidant and calcium channel blocking activity of *F. indica*. Significant restoration of ECG pattern was obtained with 100 mg/kg dose of *F. indica*. The cardioprotective action of *F. indica* was further confirmed. As ECG pattern can show uncertain patterns, estimation of serum biochemical markers can be helpful for confirming MI.

Heart contains diagnostic marker enzymes like cTnI, CPK, LDH, and AST in an abundant concentration, and it releases its content into the extracellular fluid once the heart is metabolically damaged. Significant serum level elevation of diagnostic cardiac markers (cTnI, CPK, LDH, and AST) was noticed in the isoproterenol-treated rats. Increased in the cardiac marker enzymes is the indication of isoproterenol-induced necrotic damage to the myocardial membrane which is in the line with the earlier reports [48]. Free radical generation and stimulation of lipid peroxidation by isoproterenol are the causative factor for the irreversible myocardial cell membrane damage and leakage of cardiac injury biomarkers [49].

Cardiac troponin is specific to the myocardium and considered as gold standard biomarker for diagnosing MI. As compared to troponin C (TnC) and troponin T (TnT), troponin I (cTnI) is more sensitive to cardiac injury. In MI, circulating cTnI level in the serum rises within 30 min and reaches to peak level at 18-24 h indicating myocyte apoptosis [50]. *F. indica* pretreatment significantly decreases the serum cTnI in isoproterenol-induced MI rats. It demonstrated that *F. indica* could restrict the cTnI leakage from the myocardium by maintaining structural and functional integrity and permeability of the cardiac membrane. CPK is a brain and heart muscle-specific enzyme and usually elevated as the result of myocarditis, cardiac insufficiency, arrhythmias, and myocardial infarction. Over a period of 24 h, CPK can be measured several times [51]. Isoproterenol results in the increase in serum CPK level due to the myocardial cell membrane damage and leakage of enzyme. The significant decreased in the serum level of CPK in isoproterenol-induced MI rats was observed with pretreatment with *F. indica*. Maximum decrease (41%) in the serum CPK level was observed with 100 mg/kg indicating membrane protective effect of *F. indica*.

LDH high concentration in the blood is an indicative of tissue damage and inflammatory changes in the heart. *F. indica* significantly reduces (45%) the serum LDH level in isoproterenol-treated rats. LDH is a cytosolic enzyme, which is essentially present in all the tissues involved in glycolysis [52]. LDH is not a specific marker for MI and is elevated within 24-72 h following MI and in 3-4 days reaches a peak concentration [53]. AST is a sensitive indicator of liver cell injury and is also helpful for diagnosing other diseases. In normal physiological condition, it is usually present in low concentration in the serum; however, increase is observed after MI. The probability of MI in individuals with high AST level is nearly 17 times more than the normal individuals. In 1958, AST was one of the first cardiac biomarkers used for MI diagnosis but because of weak specificity for the matter, it is no longer used as a gold standard [54]. *F. indica* significantly decreases (32%) serum level of AST. The significant fall in the serum level of cardiac markers (cTnI, CPK, LDH, and AST) indicating that *F. indica* ameliorates isoproterenol-induced cardiac injury. It demonstrated that *F. indica* could restrict these enzyme leakages by maintaining cardiac membrane integrity and permeability. However, in order to obtain the comprehensive view of the myocardial tissue architecture of isoproterenol-induced MI rats pretreated with *F. indica*, histopathological study was performed.

Histopathology provides wealth of tissue information. The preliminary histopathological examination of the isoproterenol-treated rat myocardium showed edema, inflammatory cell infiltration, necrosis, and separation of cardiac muscle fibers. Overproduction of ROS by isoproterenol can cause severe cellular function impairment and necrotic lesions in the myocardium of rats. Isoproterenol generates superoxide radicals at the site of damage and modulates superoxide dismutase, glutathione peroxidase, and catalase [55]. Pretreated groups with *F. indica* extract (100 mg/kg) showed significantly reversed edema, inflammatory cell infiltration, and necrosis as seen with the isoproterenol-treated group. Previous study has shown *F. indica* exhibits antioxidant activity by scavenging free radicals and decrease malondialdehyde (MDA), an indicator of lipid peroxidation by increase in the level of free radical scavenging enzymes such as glutathione (GSH), superoxide dismutase (SOD), and catalase (CAT) [56]. Myocardial necrosis is also detected by using stain 2,3,5-triphenyltetrazolium chloride (TTC) with which LDH of the viable myocardial tissue forms red formazan precipitate in the presence of an intact dehydrogenase enzyme system whereas the infarcted myocardium fails to stain with TTC. Consequently, TTC-unstained regions correspond to the total necrotic areas [57]. In isoproterenol-treated rats, large unstained region with more necrotic patches was observed. Significant lesser degree of unstained region was observed with pretreatment with *F. indica* in isoproterenol-treated rats showing greater tissue viability with less necrotic tissues. The maximal tissue protection was observed with 100 mg/kg dose of *F. indica*. The cardioprotective action of *F. indica* was further confirmed by its effect on rat atria.

To have insight into the effects of extracts on cardiac muscles, *F. indica* crude extract was further tested on isolated rat atrial strips. Extract completely repressed force (negative inotropic) and rate (negative chronotropic) of spontaneous atrial contractions. Thus, it is suggested that the cardioprotective effects of *F. indica* against isoproterenol-induced MI might be due to negative inotropic and chronotropic effects, which restore coronary blood supply to the myocardium. To determine the vascular effects of *F. indica*, isolated rat aortic rings were used.

When tested on vascular preparation precontracted with phenylephrine, *F. indica* extract produced complete relaxation at the concentration of 1.0 mg/ml; this relaxation was not modified with _L_-NAME a nitric oxide synthase inhibitor [58], thus excluding the role of nitric oxide pathway. However, cumulative addition of *F. indica* extract relaxed high K^+^-induced contractions at similar doses. High K^+^ increases intracellular level of Ca^+2^ from voltage-dependent Ca^+2^ channels [59]. The relaxation of high K^+^ precontraction by the extract suggests that the vasorelaxant effect possibly mediated through calcium channel blockade. These findings partly explain the negative chronotropic and ionotropic effects of *F. indica*. Previous studies have shown that alkaloids, flavonoids, and phenols exhibit cardioprotective activity [60]. Therefore, the content of alkaloids, flavonoids, and phenols reported in *F. indica* extract might be responsible for myocardial cell membrane protective activity. Protopine an alkaloid present in *F. indica* might be contributed to the cardioprotective effect and can be further investigated.

## 5. Conclusion

These findings indicate that *F. indica* possesses cardioprotective and vasorelaxant effects. *F. indica* offers protection to myocardium by maintaining ECG patterns, cardiac marker enzymes, and histopathological parameters. These effects could be due to its negative inotropic and chronotropic effects, antioxidant, and membrane-stabilizing properties. Vasodilatory effects is mediated by inhibiting calcium released and decreasing cardiac rate and force of contraction. These findings may be helpful to justify further investigations concerning *F. indica* cardioprotective effects. For further evaluation, the use of bovine coronary artery would be the better approach to have more specific insight in to the mechanisms. There is need to explore plant constituents which are responsible for its cardioprotective and vasorelaxant effects.

## Figures and Tables

**Table 1:** Qualitative estimation of cardiac troponin I in various treatment groups.

Treated group	Animal no. 1	Animal no. 2	Animal no. 3	Animal no. 4	Animal no. 5
Group 1	Negative	Negative	Negative	Negative	Negative
Group 2	Positive	Positive	Positive	Positive	Positive
Group 3	Positive	Negative	Positive	Positive	Positive
Group 4	Positive	Positive	Negative	Positive	Positive
Group 5	Positive	Positive	Positive	Negative	Negative
Group 6	Negative	Negative	Negative	Positive	Negative

Key: qualitative estimation of cardiac troponin I (cTnI) in the serum of control and treated groups. Group 1, control showed all negative cTnI test (no myocardial injury); group 2, the isoproterenol-induced MI group showed all positive cTnI test, an indicator of myocardial necrosis; groups 3 and 4 (10 and 30 mg/kg), *F. indica* extract-treated groups showed decrease in the number of positive cTnI test; group 5 (100 mg/kg), the *F. indica* extract-treated group showed significant reduced positive cTnI test; group 6, the atenolol (10 mg/kg)-treated group showed decreased positive cTnI test.

## Data Availability

Data is available on request to the corresponding author, Dr. Abdul Jabbar Shah, Email: jabbarshah@cuiatd.edu.pk.
